# A global dataset of the cost of capital for renewable energy projects

**DOI:** 10.1038/s41597-025-05912-x

**Published:** 2025-10-08

**Authors:** Bjarne Steffen, Florian Egli, Anurag Gumber, Mak Ðukan, Paul Waidelich

**Affiliations:** 1 Climate Finance and Policy Group, ETH Zurich, Zurich, Switzerland; 2 School of Social Sciences and Technology, Technical University of Munich, Munich, Germany; 3 Carbon Neutrality and Climate Change, Hongkong University of Science and Technology, Guangzhou, China

**Keywords:** Energy economics, Climate-change mitigation

## Abstract

The cost of capital (CoC) critically influences the levelized cost of renewable energy and, by extension, the global low-carbon transition. However, reliable and consistent CoC data remain scarce, limiting an appropriate reflection of CoC differences in energy system and integrated assessment models. We present a global dataset of CoC for renewable energy projects, covering 68 countries from 2010 to 2022 and focusing on three key technologies: utility-scale solar photovoltaics, onshore wind, and offshore wind. We systematically compile and standardize data from academic literature and international organizations, ensuring methodological comparability. Our dataset includes 1,429 data points, of which 366 provide nominal, after-tax weighted average cost of capital values. We conduct technical validation through cross-technology comparisons, temporal consistency checks, and source triangulation. By addressing a key data gap, this dataset aims to support evidence-based energy policy analysis and advance the understanding of how financing conditions impact renewable energy costs globally.

## Background & Summary

The global transition to energy systems aligned with the Paris Agreement’s climate targets requires substantial investments in low-carbon technologies, particularly in renewable energy technologies such as wind energy and solar photovoltaics (PV). Estimates from the academic literature suggest that these investments need to reach hundreds of billions USD annually^1–3^. These investments need to be financed, and the financing cost of energy assets, i.e. the cost of capital (CoC), critically influences the levelized cost of clean energy^4^, and the cost of the low-carbon energy transition overall^5^.

One important characteristic of many renewable energy technologies is that they are very capital-intensive: Given that they do not need fuel and only very limited maintenance work, the levelized cost of electricity (LCOE) from wind and solar depends heavily on upfront investments. This is a key difference compared to fossil fuel-based power plants, where fuel costs dominate lifetime expenditures. Accordingly, a small increase in the CoC can disproportionately raise the LCOE of renewables, undermining their competitiveness relative to fossil fuels^6,7^.

Following a common definition, the CoC represents “the expected rate of return that market participants require in order to attract funds to a particular investment”^8^. Accordingly, the CoC is closely tied to the underlying risk associated with future cash flows, accounting for systematic market-wide risks and risks that are specific to a particular technology. Importantly, the CoC for infrastructure investments—including renewable energy assets—varies widely across countries, technologies, and over time^4,9–12^. This variability has profound implications for researchers analyzing energy and climate policies. When using energy system models or integrated assessment models (IAMs), it is critical to incorporate realistic, differentiated CoC values to capture the true cost dynamics of decarbonization pathways. However, these models often rely on uniform or outdated CoC assumptions, which can distort projections of optimal technology choices, deployment rates, and policy impacts^13,14^. By oversimplifying CoC across regions or technologies, models risk misrepresenting the attractiveness of renewables, ultimately influencing policy recommendations in ways that may hinder equitable and efficient transitions^15^.

Despite its importance, high-quality data on the CoC remain scarce. Project developers and financiers typically regard their financing conditions as competitive information and keep them proprietary^16^. Nevertheless, two strands of data emerged over the last decade: First, academic researchers have developed empirical methods to estimate CoC, ranging from the elicitation of project finance deal information to econometric analyses of financial market data. An earlier meta-analysis by Steffen^11^ compiled academic CoC estimates up to 2017 (published in articles published up to 2018) but did not capture the substantial growth in this research area over the subsequent five years, nor could it account for major global economic disruptions after 2017 impacting global interest rate levels such as the COVID-19 pandemic^17^. Second, international organizations (IOs), namely the International Energy Agency (IEA) and the International Renewable Energy Agency (IRENA), have recently organized surveys to better understand the financing conditions of renewable energy projects in specific countries.

While these initiatives mark significant progress, the data for renewable energy CoC remains piecemeal, with information distributed across many different sources, and values often not directly comparable due to inconsistencies in methodologies, particularly tax treatments. This limits the applicability for cross-country or multi-technology studies—which are at the heart of many energy system models, and even more so for IAMs. There is no authoritative data source that modelers could draw on. This lack of a broad and consistent CoC dataset likely contributes to the common practice of ignoring CoC heterogeneity altogether^13^.

In response to these shortcomings, this study presents a broad and consistent dataset on the CoC for renewable energy projects across a wide range of countries, spanning the 2010–2022 period. We systematically integrate and standardize available data for three key technologies—utility-scale solar PV, onshore wind, and offshore wind—resolving discrepancies in tax treatment and other methodological differences. Compared to previous research, this dataset particularly covers a longer timeframe (five more years than the only available prior meta-analysis^11^), a substantially higher number of data points (1,429 data points here vs. 358 in ref. ^11^), and for the first time brings together evidence from academic peer-reviewed studies and data from International Organizations in a systematic way. By offering a ready-to-use dataset, we aim to facilitate more accurate modeling in energy system and IAM frameworks, while also informing public policy analyses that seek to reduce financing barriers for renewables. Ultimately, this work aspires to advance understanding of how financing conditions shape the global low-carbon transition, supporting evidence-based energy policy advice.

## Methods

**Fig. 1:**
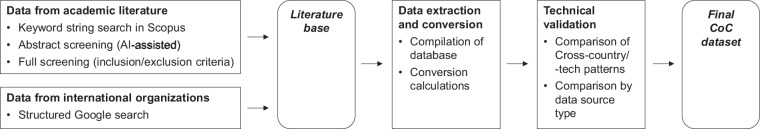
Workflow of the construction of the final CoC dataset.

Our study conducts a meta-analysis of CoC data, based on a systematic review of original research (from academia and from IOs) that quantifies the CoC for renewable energy projects. The workflow is described in Fig. 1.

First, we compile relevant literature from two sources: On the one hand, academic peer-reviewed articles—using the existing meta-analysis from Steffen^11^ for data from articles published until 2018 and using their methodology for articles published 2019–2023. Unlike in the former meta-analysis, in this study we utilize a machine-learning aided framework (ASReview), given the increased number of published articles. On the other hand, in this study we newly add a non-academic data source, namely data collected by IOs. Given the call for more CoC data transparency inter alia from previous academic literature, in recent years actors like the IEA conducted targeted studies, which can add further data to our analysis.

Second, we analyze the identified studies in detail, extract all relevant CoC information (typically numerous variables per study, as described below), and conduct calculations to ensure comparability of the data, using additional data sources where necessary (e.g., concerning tax rates).

Third, we compile a standardized dataset and conduct a number of descriptive analyses for technical validation, triangulating the plausibility of CoC data by comparing patterns between countries across technologies, consistency over time, and between data from different data sources (academic studies vs. IOs). The individual steps are described in detail below.

### Scope

Our dataset comprises the CoC for commercial projects in three renewable energy technologies, namely utility-scale solar PV, onshore wind turbines, and offshore wind turbines. These technologies make up the lion’s share of capacity additions globally^18^. We consider CoC values from 2010 on (i.e., starting after the Global Financial Crisis 2008–09), and up until 2022 as per data availability as we include articles and studies published until 31 December 2023 (given the usual duration of academic publishing processes, the most recent estimates in these studies are typically for the year before publication). Our scope is global, including all countries for which data is available (see below). Concerning the academic articles, it should be noted that similar to the approach in Steffen^11^, we include all studies that provide sufficiently detailed and rigorous empirical data on CoC, irrespective of whether providing this data was the primary goal of the study or not (e.g., the CoC was estimated to calibrate model parameters).

### Data compilation

In this study we combine (as far as we know for the first time) data from academic literature and from IO reports. While grey literature, such as IO reports, typically does not undergo a rigorous peer-review and often is less transparent concerning underlying methodologies, adding this data source allows for substantially extending the available data. In the case of CoC estimates, specifically, IOs such as the IEA and IRENA also have unique access to providers of original data that academic researchers have not, which further speaks for including their data. A comparison of estimates from academic vs. IO data sources is presented below as part of the technical validation.

#### Academic literature

**Fig. 2:**
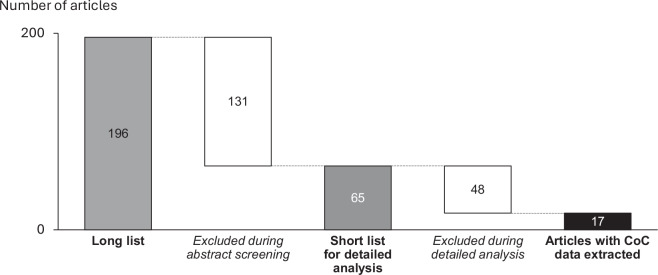
Literature funnel for academic studies published 2019–2023. Earlier studies are included as per the meta-analysis in ref. ^11^.

We apply the protocol for a systematic and reproducible compilation of the literature base as described in ref. ^11^, which follows common standards in social sciences^19,20^. The literature funnel is illustrated in Fig. 2: Applying the search string

*TITLE-ABS-KEY (("cost of capital" OR "financing cost" OR "financing conditions" OR ("risk premium" AND "investment") OR ("capital markets" AND "investment")) AND ("renewable energy" OR "clean energy" OR "sustainable energy" OR "solar" OR "wind")) AND PUBYEAR* *>* *2018 AND PUBYEAR* *<* *2024*

to the abstract database Scopus, we get a list of **196** articles. For these articles, meta-data was extracted and loaded into ASReview, an open-source machine learning tool for abstract screening in meta-analyses, developed by a team at the University of Utrecht^21^.

The ASReview tool helps in the abstract screening by continuously re-ordering abstracts according to their expected relevance as per an active learning model (using previous classifications as an input). While in our case all 196 abstracts have been screened by researchers (human-in-the-loop), the machine learning-aided approach allows an efficient screening where attention is focused on the more relevant articles, an increasingly common approach for systematic reviews and meta-analyses^22^. For the active learning model, we choose the ASReview default parameters, namely Naive Bayes as classifier, Max as query strategy, and tf-idf for feature extraction. The short list of papers likely fulfilling our inclusion criteria as per the scope defined above included **65** articles.

For the short list, all articles have been analyzed in detail whether they provide sufficient data to quantify after-tax weighted average cost of capital (WACC) for a specific country, year and technology, and whether the estimation method is sufficiently clearly described. For those articles fulfilling these criteria, applicable data is then extracted and used to calculate comparable CoC values as described below. Eventually, after-tax WACC values could be calculated based on data extracted from **17** articles published during 2019-2023, adding to the data from 14 articles studied before 2019 that were analyzed in ref. ^11^. Taken together, sources from academic literature are refs. ^4,7,9,10,16,23–57^.

#### Data from international organizations

Implementing a reproducible literature search in grey literature (such as IO reports) is generally challenging as there is no central/fixed database of this research. However, in our case the list of potential international organizations is limited because of the specificity and technical nature of our research scope. Relevant domain-specific organizations are the IEA and IRENA—some authors of this study have been involved with both in setting up studies on CoC for renewables, underlining the relevance of these organizations. Second, multilateral development banks could be relevant given their renewable energy financing activities, namely the World Bank Group and nine regional development banks: Asian Infrastructure Investment Bank, African Development Bank, Asian Development Bank, Development Bank of Latin America, European Bank for Reconstruction and Development, European Investment Bank, Inter-American Development Bank, Islamic Development Bank, and the New Development Bank as discussed in Steffen & Schmidt^58^.

For all these organizations, we conducted a google search for “cost of capital for renewable energy + [org]”, “financing cost for renewable energy + [org]” and “financing conditions for renewable energy + [org]”, considering ten pages of results, followed by a screening of the data offered via the organization’s own data portal if available. We also held informal discussions with experts from several of these organizations about whether we overlooked any potential data source. In sum, there were just two (though extensive) data sources from IOs: The IEA’s Cost of Capital Observatory^59^ and IRENA’s report on financing cost for renewable energy^60^. Concerning the IEA, the full underlying database is available online where it could be extracted; concerning IRENA, the authors obtained the detailed data directly from the organization (datapoints are also available in charts shown in the report PDF).

### Data extraction and conversion

There are numerous different CoC measures^8,11,61^. For policy analysts and modelers aiming to simulate market outcomes, the most relevant measure is the private CoC after tax effects, i.e. the “price that profit-maximizing capital providers demand for issuing debt (e.g., loans) or equity”^13^. Specifically, we aim to calculate the nominal after-tax WACC, that is

$${{WACC}}_{{after}-{tax}}={DS}\left(1-\tau \right){CoD}+\left(1-{DS}\right){CoE},$$

Where *DS* is the debt share, *CoD* is the cost of debt, *CoE* is the cost of equity, and τ is the corporate tax rate. The WACC_after-tax_ is the appropriate discount rate for economic appraisals, such as in net present value calculations, in intertemporal optimization models that simulate market outcomes, and for levelized cost comparators such as LCOE^13^.

For our dataset, we extract all individual CoC components and aggregated WACC_after-tax_ values per country, technology, and year, alongside information of the underlying estimation method. Where more than one value per country/technology/year is available for CoC components (e.g., because the original study provides a list of projects with CoC data for each of them), we calculate an average and indicate in a comment on how many data points the average is based. In case the WACC_after-tax_ is not provided, we calculate it based on the data for CoC components given in the study. In numerous cases, no after-tax WACC is provided but rather a pre-tax WACC or a “vanilla” WACC that abstracts entirely from tax considerations. In these cases, we calculate the after-tax WACC using additional data on the applicable corporate tax rate in the given country and year based on KPMG^62^ or PWC^63^, as explained in a comment in the database. The resulting dataset clearly indicates what are empirical values, and what are additional assumptions or values that are calculated by the authors for the meta-analysis.

### Data coverage

**Fig. 3:**
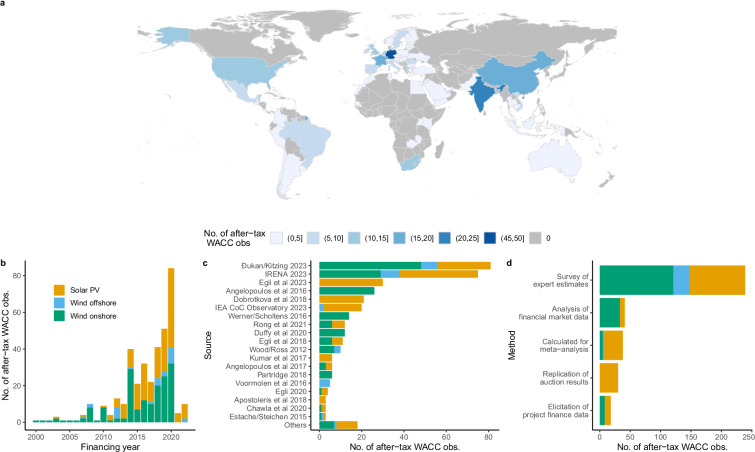
Overview data coverage.

In total, our dataset covers 1,429 data points (WACC, cost of debt, cost of equity, etc.), thereof 366 nominal WACC_after-tax_ values. Figure 3a illustrates the geographies covered: In total there are WACC values available for 68 countries, covering all world regions. The coverage seems to roughly align with renewable energy capacity deployment, having more info on China, Europe, the Americas, and little on Central Asia or Sub-Saharan Africa (except South Africa). We like to iterate that this coverage fully depends on the availability of primary research – the data gap in some regions should certainly be addressed by additional primary research in the future^64^.

Concerning the availability of data over time, Fig. 3b shows that most data points refer to financing years from 2014 on, with onshore wind CoC being available first, then solar PV CoC from about 2012 on, and offshore wind mostly from 2018 on (which is in line with the deployment and hence investment patterns in these technologies^64^). The peak in 2020 with 84 data points is driven by the IRENA data referring to this financing year, alongside many academic studies that came out in 2022 and 2023. The low number of data points in 2021 and 2022 is likely due to our cut-off date for literature to be included (Dec 31, 2023).

Our dataset combines information from 32 primary studies. Figure 3c shows the contribution of individual data sources. The top 5 include Đukan & Kitzing^38^ with data for all technologies from a cross-European research project, IRENA^60^ with data from a global IO study spanning all technologies, Egli *et al*.^37^ with data for solar PV in nine countries over time, Angelopoulos *et al*.^9^ with data for onshore wind and solar PV from a cross-European research project, and Dobrotkova *et al*.^10^ with data for solar PV across thirteen developing countries. The figure also shows that many studies span multiple technologies.

Concerning the underlying estimation methods, Fig. 3d illustrates that the majority of original studies rely on surveys of expert estimates (65% of data points), followed by analyses of financial market data (11%), a replication of auction results (8%), and the elicitation of specific project finance data (5%). About 10% of data points are WACC values that have been calculated for the meta-analysis based on CoC components in the original studies and further assumptions.

**Fig. 4:**
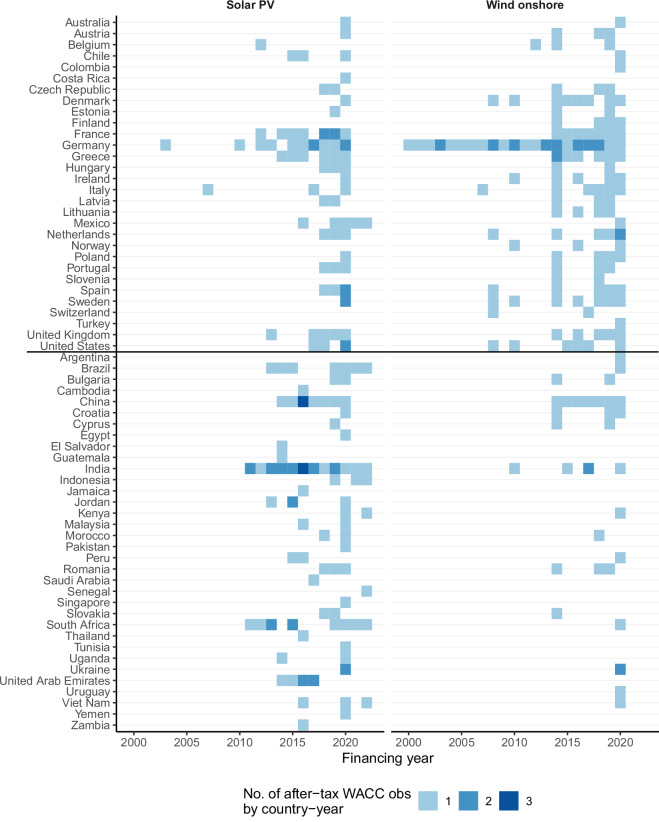
Heat map of WACC observations for solar PV and onshore wind.

**Fig. 5:**
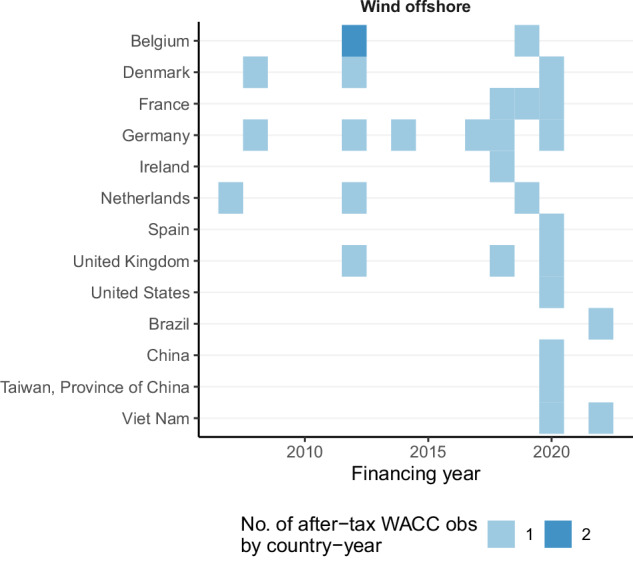
Heat map of WACC observations for offshore wind.

The heat maps in Fig. 4 and Fig. 5 provide a more granular illustration of data availability. Generally, the coverage is better for OECD countries than non-OECD countries (apart from large emerging economies). Many countries feature just 1–3 data points per technology over the years. In the OECD, more than three years are covered for Denmark (onshore wind), France (solar PV and onshore wind), Germany (solar PV, offshore wind, and onshore wind), Greece (solar PV and onshore wind), Ireland (onshore wind), Italy (onshore wind), Lithuania (onshore wind), Mexico (solar PV), Netherlands (onshore wind), Poland (onshore wind), Span (onshore wind), Sweden (onshore wind), United Kingdom (solar PV and onshore wind), and the United States (onshore wind). In non-OECD countries, data for more than three years is available for Brazil (solar PV), China (solar PV and onshore wind), India (solar PV and onshore wind), South Africa (solar PV), and the United Arab Emirates (solar PV). As highlighted by Fig. 4 and Fig. 5, there are very few country/technology/year combinations where CoC data points from more than one study are available, and not a single country/technology/year has more than three CoC values. This lack in overlap of studies suggests that a technical validation of the compiled dataset requires comparing data points across years or across technologies (see below) and also calls for further primary research to improve the robustness of CoC estimates.

## Data Records

The final dataset is available on *figshare*: Steffen, Bjarne; Egli, Florian; Gumber, Anurag; Ðukan, Mak; Waidelich, Paul: A global dataset of the cost of capital for renewable energy projects. figshare. 10.6084/m9.figshare.28588943 (2025)^65^. The following information is included:

### Study type

Indicator whether the original study from which the datapoint stems is an *academic peer-reviewed study* or an *international organization report*.

### Study short name

Brief name of the original study from which the data point stems, e.g. “Partridge 2018”.

### Study location

Identifier of the original study from which the data point stems. For academic studies the Digital Object Identifier (doi), for international organization reports the web address (URL).

### Country code ISO2

The iso-2 country code for the country to which the data point refers.

### Country code ISO3

The iso-3 country code for the country to which the data point refers.

### Country name

The name of the country to which the data point refers.

### Country group

Indicator whether country is part of the Organization for Economic Cooperation and Development (*OECD*) or not (*non-OECD*).

### Technology

The technology to which the data point refers, namely *solar PV*, *wind offshore*, or *wind onshore*.

### Variable

The variable name of the data point, namely *Cost of debt, Debt margin on gov bond, Debt margin on LIBOR, Leverage (debt share), Return on equity, Tax Rate, WACC (nominal after-tax), WACC (nominal pre-tax), WACC (nominal vanilla), WACC (real after-tax), WACC (real vanilla).*

### Value

The value of the data point, in %.

### Type of data

The nature of the data point, namely *assumption in article, empirical result, value calculated for meta-analysis.*

### Estimation method (category)

The estimation method in the original study, namely *Analysis of financial market data*, *Elicitation of project finance data*, *Replication of auction results*, *Survey of expert estimates*. See Steffen^11^ for a detailed discussion of the different methods.

### Estimation method (details)

Additional details on the estimation method used in the original study, where applicable.

### Comment on calculation for meta-analysis

Additional information for the calculation performed as part of the meta-analysis, like additional assumptions used, where applicable.

## Technical validation

### Overview

The meta-analysis combines information from 32 primary research studies that rely on numerous different estimation methods and differ in how detailed the underlying method and source data is described. Further, in some cases additional assumptions are needed to calculate comparable values (see section 2.3). These factors could lead to inconsistent or implausible data points. Following common practice in meta-analyses, we address these potential challenges by (1) a careful process for data compilation and standardization, and (2) several consistency checks.

Concerning the process for data compilation and standardization, all potentially relevant studies have been reviewed in detail by a researcher with ample relevant experience – all authors have previously published empirical academic work on the CoC for renewables^4,11,37,57,66–69^. We only included data from studies where the CoC components were clearly defined and the estimation method was described sufficiently clearly (i.e., allowing to classify the underlying methodology in the dataset). Any ambiguities were discussed with a second researcher, and where necessary, clarifications with the authors of the original studies were sought. All entries have then been checked for plausibility by a second researcher.

Concerning consistency checks, based on the data coverage three analyses could be conducted: Comparing the pattern of country differences across the three technologies, scrutinizing the development over time for technology-country pairs, and considering the consistency between data sources.

### Consistency of country differences across technologies

**Fig. 6:**
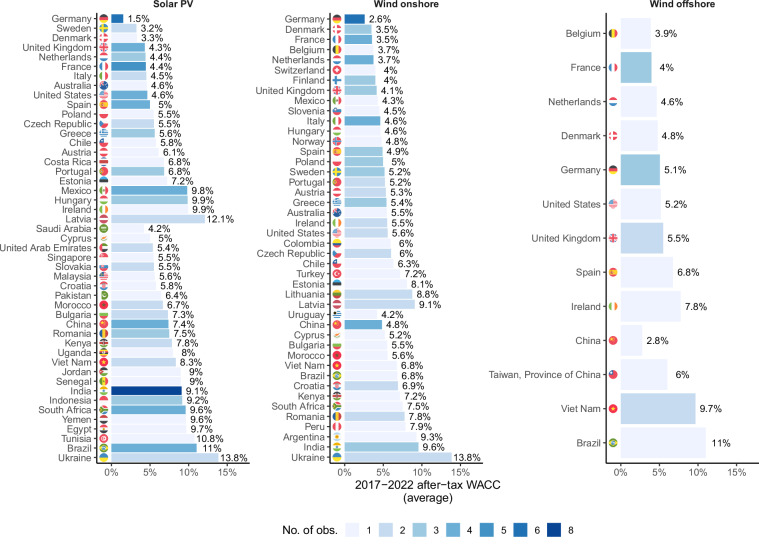
WACC estimates per country across technologies.

Clearly, there are substantial differences between countries concerning the CoC. Further, the CoC for renewable energy projects (like for other assets) vary over time, due to fluctuations in the general interest rate level^7^, and changes in the maturity of technologies^4^. In our dataset, the last six years (2017–2022) that comprise much of the data points, however, were characterized by relatively low general interest rate levels across world regions, and both solar PV and onshore wind being mature technologies in many countries. Accordingly, in a consistent dataset we would expect a certain (though not complete) correlation between different technologies’ average CoC in the same country. Figure 6 shows the average CoC per country for all three technologies, ordered by magnitude of the CoC for solar PV, showing a clear pattern between countries. As expected, in most cases we see that countries having a comparably low CoC for solar PV also have a comparably low CoC for onshore wind (given that both variables are available) and so on, underlining the consistency of the dataset with respect to this matter. A notable exception is China, where the CoC for offshore wind is much lower than for the other technologies - primarily because the only available data point for Chinese offshore wind comes from IRENA, whose CoC estimates for China are generally lower than academic estimates. Overall, the correlation between the CoC for solar PV and onshore wind is 75.3%, between solar PV and offshore wind 69.3%, and between onshore wind and offshore wind 77.8%. In sum this test suggests a generally consistent dataset.

### Within-country consistency over time

**Fig. 7:**
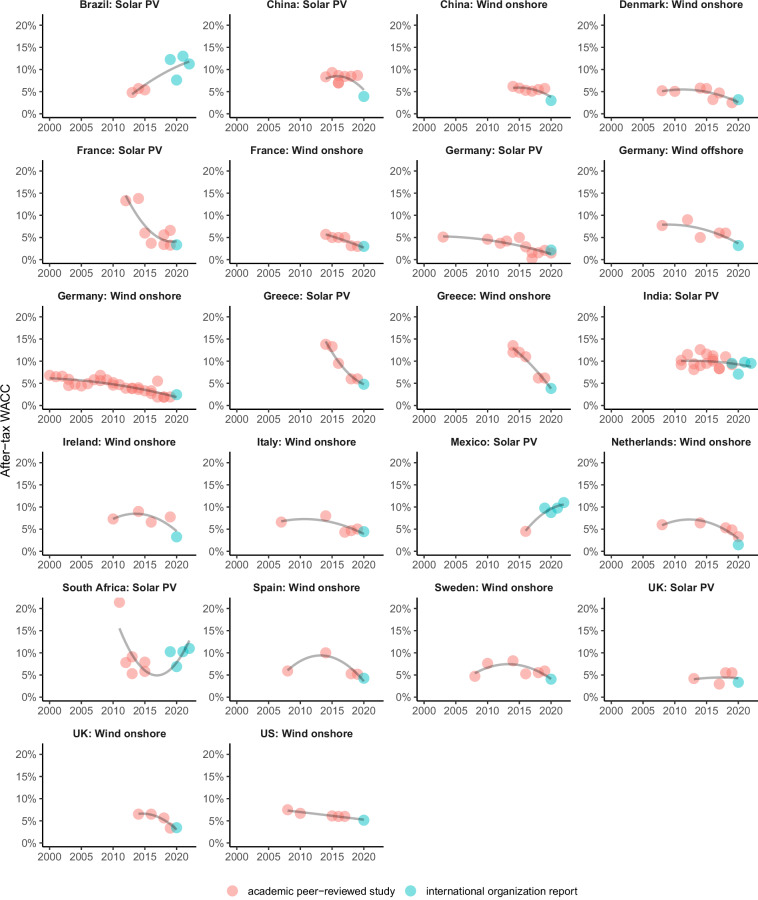
WACC estimates for solar PV and wind onshore over time (only countries with data for at least five years). Each dot represents one data point.

For a specific country-technology pair, we can also analyze the development over time for consistency. Figure 7 shows WACC estimates over time for those countries where data for at least five years is available. In most cases, we see a general decline over the time span covered, which is consistent with the trend of declining general interest rate levels and technologies becoming more mature^4^. For the large majority of countries, the development is relatively smooth, which speaks for consistency in the dataset. There are some noticeable exceptions, though: In Spain, there is a massive increase after 2012, where a generous support policy has been changed retroactively, shattering international investor confidence in the market and leading to large risk premia for some time. In Greece, CoC have been very high following the sovereign debt crises, before they declined substantially in recent years. These country-specific patterns can be explained by the fundamental reasons mentioned, so they do not speak against the consistency of the dataset. Some other outliers, however, cannot easily be explained, such as the very high CoC in early years in France and one outlier in South Africa, and a very low CoC in Mexico in an early year.

### Consistency between data sources

In this dataset, we newly combined CoC estimates from academic peer-reviewed studies with data from IO reports. As a consistency check, Fig. 7 also highlights the difference between estimates from different data sources. For Denmark (wind onshore), France (solar PV and wind onshore), Germany (solar PV, wind offshore, and wind onshore), Greece (solar PV and wind onshore), India (solar PV), Italy (wind onshore), Italy (Wind onshore), Netherlands (wind onshore), South Africa (solar PV), Spain (wind onshore), Sweden (wind onshore), UK (solar PV and wind onshore), and the US (wind onshore) both data sources are very consistent. Only for China (solar PV and wind onshore) we see the data points from an IO report (IRENA in this case) to be much lower than the academic data. For Brazil (solar PV) and Mexico (solar PV), we see the IO data points much higher than academic data, though the latter dates from several years back. It should be noted that the consistency check between data sources is only possible for more recent data points, as the IO surveys do not date back to before 2019. In earlier years, a number of academic estimates reported much higher CoC (e.g., in France or Greece), and we do not have IO estimates for comparison.

In sum, we conclude that the technical consistency checks show a high consistency of the CoC dataset, despite the multiple different data sources and underlying estimation methods. Some outliers suggest that relying on individual data points might require special caution and more primary research is needed to explore potential reasons for systematic discrepancies, such as the divergence of IO-based and academic estimates for Chinese CoC. Nevertheless, we believe that the broad dataset can serve as an important resource for model-based analysis of renewable energy investment, and the low-carbon energy transition more broadly.

## Data Availability

No customized code was produced to prepare the dataset. All scripts to reproduce the figures in this paper are available at 10.5281/zenodo.16631774.
